# The current state and potential innovation of fetal cardiac MRI

**DOI:** 10.3389/fped.2023.1219091

**Published:** 2023-07-14

**Authors:** Michelle Udine, Yue-Hin Loke, Suma Goudar, Mary T. Donofrio, Uyen Truong, Anita Krishnan

**Affiliations:** Division of Cardiology, Children’s National Hospital, Washington, DC, United States

**Keywords:** fetal cardiac MRI, fetal MRI, fetal ultrasound, 4D flow, prenatal diagnosis of congenital heart disease, fetal echocardiography

## Abstract

Fetal cardiac MRI is a rapidly evolving form of diagnostic testing with utility as a complementary imaging modality for the diagnosis of congenital heart disease and assessment of the fetal cardiovascular system. Previous technical limitations without cardiac gating for the fetal heart rate has been overcome with recent technology. There is potential utility of fetal electrocardiography for direct cardiac gating. In addition to anatomic assessment, innovative technology has allowed for assessment of blood flow, 3D datasets, and 4D flow, providing important insight into fetal cardiovascular physiology. Despite remaining technical barriers, with increased use of fCMR worldwide, it will become an important clinical tool to improve the prenatal care of fetuses with CHD.

## Introduction

While fetal echocardiography remains the first line to diagnose cardiovascular anomalies in-utero, MRI has increasingly been used as a complement to ultrasound-based modalities. Fetal echocardiography was first introduced in the mid-1980s (1) and soon thereafter became the mainstay for assessment of the fetal cardiovascular system, as it is safe, widely accessible, and highly accurate in the diagnosis of congenital heart disease (CHD) (2). There are limitations to fetal echocardiography, however, that includes unclear imaging due to maternal body habitus, fetal position, fetal bone ossification, oligohydramnios, and a limited field of view (2, 3). In addition, there continues to be some assessments that remain elusive, including accurate diagnosis of congenital defects such as coarctation of the aorta that may be better appreciated with acquisition of blood flow characteristics. Acquisition of ventricular volume and flow data would be useful to better quantify valve regurgitation and ventricular function to determine the degree of cardiovascular compromise in fetal conditions at risk for hydrops fetalis. Therefore, there is a clinical need for a complementary imaging modality to improve current prenatal care for CHD and the fetal cardiovascular system.

In contrast to other organ systems, assessment of the fetal cardiovascular system requires dynamic imaging to resolve cardiac motion and blood flow. Fetal cardiac MRI (fCMR) to date has been technically limited due to fetal motion, small size of fetal structures, and lack of a real time fetal electrocardiogram (ECG) to synchronize data acquisition to the fetal cardiac cycle. Innovative technology has allowed some of these barriers to be overcome and increased the potential for application of fCMR clinically (4). In addition, there have been improvements in acquisition techniques allowing for improved image quality, acquisition speed, and correction of motion artifact in both anatomic and flow assessments. MRI techniques including radial undersampling and compressed sensing have allowed for creation of motion corrected cine reconstructions (5–9). fCMR is a promising supplementary imaging modality to fetal echocardiography for the diagnosis of CHD in weeks 30–40 of pregnancy. The ability of MRI to assess blood flow and 3D volumes and application of this technology to the fetus may provide new insight into fetal cardiac physiology and development. Important challenges remain including the cost, long scan time, and need for experienced clinicians to acquire and interpret each study. Few centers across the world are utilizing this technology, with fCMR in the early phases of clinical implementation with practical obstacles to overcome (10).

This paper will review the state-of-the-art of fCMR, including new technologies instituted in recent years, methods and workflow of fCMR, safety, current clinical applications of fetal MRI and fCMR, and opportunities for innovation.

## Challenges in fetal CMR gating techniques and advancements of fetal ECG

**Figure 1:**
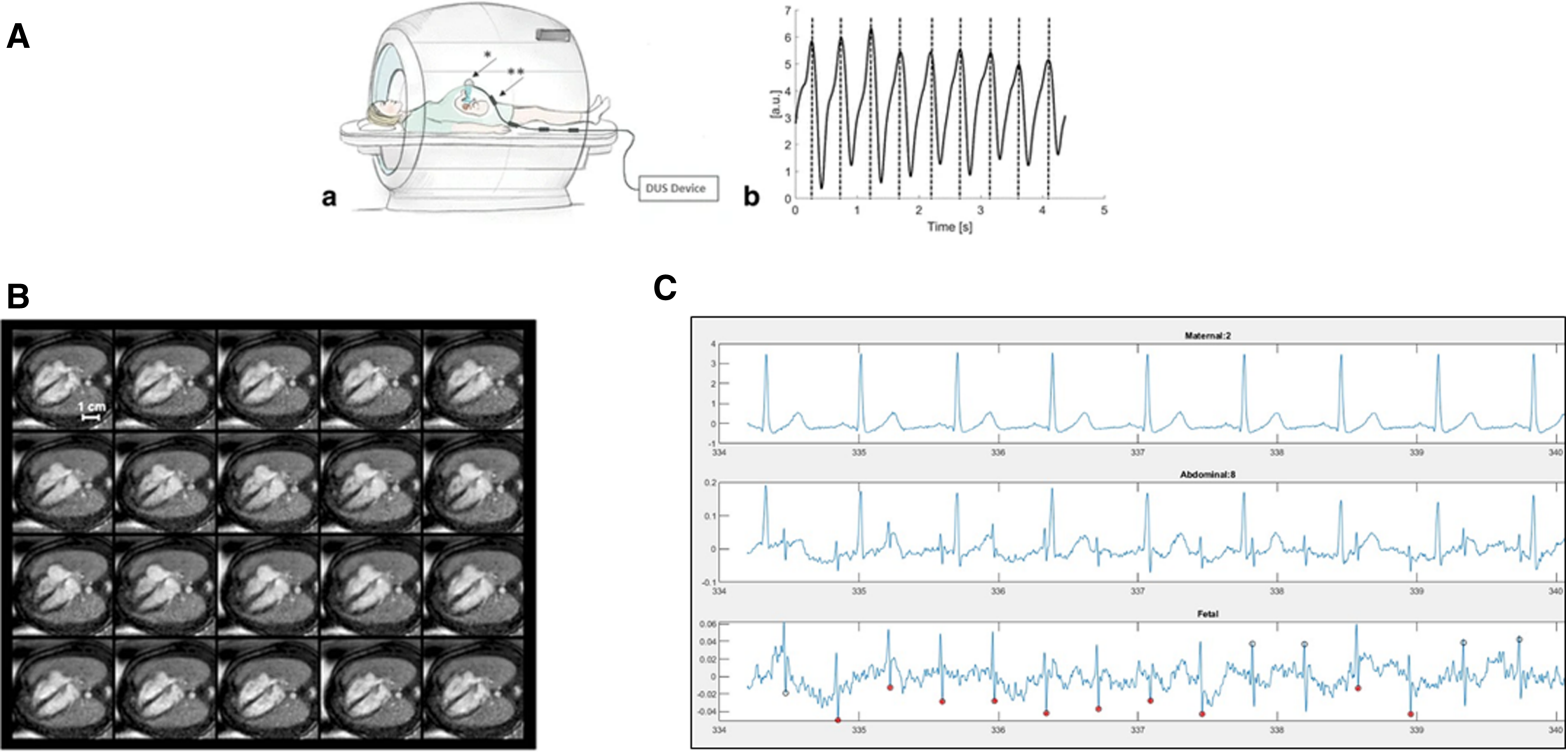
(**A**) Doppler ultrasound trigger device. (**a**) Schematic illustration of the experimental setup during fetal CMR showing placement of the Doppler ultrasound (DUS) transducer (*) on the maternal abdomen. The connecting cable has four traps (**) to avoid electromagnetic interferences from radiofrequency pulses. (**b**) Example of generated DUS gating signals represented by maximum signal peaks. The fetal heart beat was recorded by the DUS transducer and processed to allow for maximum peak detection. Figure reprinted with permission from Kording et al., (12). http://creativecommons.org/licenses/by/4.0/. No changes were made to the figure. (**B**) Multiple phases of DUS Gated Cine Images. DUS-gated balanced SSFP cine images (20 phases) of the fetal heart in the 4-chamber view (gestational week 36). DUS gating allowed for clear differentiation of the myocardium versus lumen throughout the cardiac cycle. Symmetric contraction of the ventricles and expansion of the atria is seen with maximum ventricular contraction and minimum ventricular blood volume in cardiac phases 10–12. Figure reprinted with permission from Kording et al., (12). http://creativecommons.org/licenses/by/4.0/. No changes were made to the figure. (**C**) Fetal electrocardiogram tracing in a 26 weeks of gestation fetus. (**a**) Maternal ECG tracing (**b**) combined abdominal signal showing larger maternal and smaller fetal tracings (**c**) separated fetal ECG showing clear R waves.

There are inherent challenges of motion, cardiac gating, and achieving adequate spatiotemporal resolution that have limited the clinical use of fCMR (11). Cardiac motion can be addressed with appropriate gating. The gold standard for gating in pediatric cardiac MRI is surface electrocardiography (ECG). There are two current alternatives commonly being used in fCMR—metric optimized gating and Doppler ultrasound gating. In metric optimized gating, data is temporally over acquired and utilizes a postprocessing technique to identify the correct heart rate after scanning (5, 11). More recently, MR-compatible Doppler ultrasound (DUS) probes have been developed as an alternative to ECG for gating the fetal heart rate, as seen in Figure 1A to produce dynamic cine images shown in Figure 1B. The device provides triggers based on Doppler waveforms of the heart rate and is connected directly to the MR scanner for data synchronization. This method is subject to technical issues with re-positioning of the device necessary if the fetus moves out of the field of the transducer (13).

While fetal electrocardiogram devices have been available for a century as research tools, electrocardiography in the fetus has not yet become a part of routine clinical care. Prior work has required intensive signal averaging (14) or had significant delays between real and visualized fetal heart signals. Challenges include increased maternal body habitus, insulation of the fetus with vernix coating, fetal movement and low signal to noise ratio (15). Advances in fetal electrocardiography (fECG) in the last few decades have brought us closer to a time resolved, “beat to beat” fECG, as shown in Figure 1C. With attention to signal collection strategies and appropriate timing during gestation, it is possible to obtain clear time resolved fECG. Rapid advances in technology and processing power have the potential to overcome and facilitate current challenges in gating for fCMR (16).

## Methods and workflow of performing fetal cardiac MRI

fCMR has been successfully performed using both 1.5 and 3 T MR scanners (13). Fetal CMR is typically reserved for the third trimester after 30 weeks gestation, when the fetus is larger with fewer movements. When using the DUS device for cardiac gating, the MR-compatible DUS transducer is secured on the maternal abdomen after finding the position with the most consistent fetal cardiac signal. The device is held in place with an elastic belt. The DUS is connected directly to the CMR scanner, which can employ an external triggering using the DUS signal as a gating signal for the fCMR (13).

The most common sequences utilized for static imaging of the fetal heart include balanced steady-state free precession (bSSFP) and single-shot fast spin echo or “black blood” sequences including half-fourier acquisition single-shot turbo spin echo (HASTE) and single shot fast spin-echo (SS-FSE). b-SSFP allows for contrast between the blood pool (hyperintense) and myocardium (hypointense). The fast spin echo sequences produce T2 weighted images with black blood contrast that provide excellent contrast between the vasculature and lung/thymus (2). These sequences allow for excellent visualization of gross anatomy, mediastinal vascular anatomy, and extracardiac abnormalities (3). The benefits of these sequences include high spatial resolution (∼1.0–1.5 mm in-plane) with a short acquisition time (>500 ms) but are subject to artifact from cardiac motion. However, the static images do not provide information on detailed intracardiac anatomy, ventricular function, or valve functionality (5).

Time resolved (CINE) bSSFP imaging can be utilized with gating to obtain dynamic high spatial and high temporal resolution images (∼1 mm × 1 mm, <50 ms) that provide an assessment of both cardiac structure and function. Acquisition time of each slice of gated bSSFP cine imaging is a few seconds, making it susceptible to both fetal motion and maternal breathing (3).

To minimize artifacts from maternal respiration, maternal breath holds or shallow maternal breathing has been utilized with success (17). However, there have also been free-breathing methods developed that use motion correction to suppress artifacts enabling multi-slice acquisitions (5). There are other automated methods that have been implemented using a statistical outlier-rejection method that was previously used for fetal brain volumetric reconstruction. With outlier rejection, inconsistent data is rejected that reduces artifact and produces sharper images (5).

There is no sedation or contrast used for fetal CMR. After a scout acquisition, a series of static images in multiple planes (axial, sagittal, and coronal) are obtained. Dynamic images using gating with cine b-SSFP sequences in multiple planes (axial, sagittal, coronary, short-axis, and four-chamber views) are obtained for each fetus. Multi-slice imaging to provide whole heart coverage using parallel overlapping slices in three orthogonal planes or single-slice imaging in specific imaging planes of interest can be utilized.

The typical scanning time studies have reported are 30–60 min; however, placement of the DUS device and finding a stable fetal heart rate signal often adds time to the scan. CMR localizer sequences or fetal echocardiogram images can be used prior to the scan to help visualize the position of the fetus and fetal heart prior to DUS device placement. Some centers have successfully utilized imaging post processing techniques to produce higher resolution images (6). Depending on the goal of the study and access to post-processing software, a significant amount of time can be spent on post-processing techniques. One study successfully used an open-source imaging processing software to create high resolution 3D visualization of the fetal heart using a motion-corrected slice-volume registration from non-gated 2D MRI images. They report 30 min of total MRI time and 30 min of postprocessing time to produce 3D volume rendered images of the fetal heart (6). Accurate interpretation of fetal cardiovascular MRI should be performed by an experienced team of cardiology and radiology experts (10).

## 2D Phase contrast MRI

Phase-contrast MRI (PC-MRI) is a powerful tool that has allowed for non-invasive quantification of blood flow and is the gold standard for the hemodynamic assessment postnatally in children with congenital heart disease (18) With the technology of metric optimized gating, PC-MRI has been shown to be feasible to measure flow in major vessels in the fetus late in gestation (18). PC-MRI has been shown the flow distribution of the normal human fetal circulation with success (18, 19). There are preliminary reference ranges for late gestation human fetuses that are consistent with measurements made in fetal lambs in prior studies (18). PC-MRI has been used in combination with oximetry measurements to show many expected changes in fetal circulatory physiology in fetuses with CHD including a reduction in ascending aortic flow and increase in flow across the main pulmonary artery and ductus arteriosus in fetuses with hypoplastic left heart syndrome (20). Limitations to this technique include vulnerability to movement artifact earlier in gestation, only localized flow information at the plane of interest, and a long acquisition time to measure multiple vessels to provide a more comprehensive flow distribution assessment (18). 4-Dimensional Flow MRI (4D flow) is an appealing technology to provide a comprehensive blood flow quantification in a single acquisition.

## 4D Flow MRI

Beyond 3D anatomy and cine functional assessment, cardiac MRI allows for three-directional velocity encoding (4D flow) to comprehensively analyze vascular and valvar flow in the fetal heart. 4D flow provides qualitative dynamic blood flow imaging by streamline mapping or particle tracing to visualize phasic flows such as ductal shunts, atrial shunts, and venous flows (21, 22). The simultaneous cine and flow components in a 4D flow dataset also allow for blood flow quantification at multiple levels in a single acquisition instead of multiple acquisitions (19, 23). Both components have been applied to fetal imaging in animal trials and pilot studies (24–27). Dynamic blood flow imaging of sheep fetuses has confirmed the “spiraling” effect of umbilical vein acceleration through the ductus venosus into the foramen ovale (24). For humans, Doppler-ultrasound gating has now enabled simultaneous time-velocity/flow curves quantification of the aorta, main pulmonary artery, and ductus arteriosus in normal fetuses (27).

At the same time, considerable technical challenges remain, accounting for a considerable failure rate (∼25%) in feasibility studies (27). 4D flow is sensitive to magnetic eddy currents, has limited spatial/temporal/velocity resolution and significant potential for respiratory/motion artifact, all of which affect the derivation and calculation of flow parameters (28, 29). There continues to be ongoing development of 4D flow sequences specific to the fetal heart, such as slice-to-volume registrations combined with velocity-encoding (covering the entire heart in a series of non-coplanar stacks) (30, 31). Other general developments to 4D flow will also be advantageous for fMRI, such as incorporation of compressed sensing (32) that could reduce scan time. Machine learning is in the early stages of development with a goal to incorporate expected fluid behavior (as derived by computational fluid dynamic simulations) and enhance the resolution of 4D flow imaging (33). These developments will help reduce the uncertainties behind measuring flows in small vessels, in a non-sedated fetus with an inherent fast heart rate.

The advantage of 4D flow is that it offers the potential for a “true” 3D representation of fetal flow, allowing for *in-vivo* investigations of flow characteristics governing heart remodeling/disease progression that were previously limited to computational methodologies (34). Intriguing hypotheses such as the significance of flow mediated factors in developing HLHS could be addressed as one example. Flow phenomena previously visualized qualitatively by echocardiography could potentially be quantified and used to guide therapy, such as comprehensive flow/shape quantification of the aorta and ductus arteriosus to improve the sensitivity of detecting aortic coarctation (35), or flow markers to quantify the effect of CHD on the fetal cerebral circulation (36). As technical developments continue to enhance 4D flow, there will be a role for improved flow visualization, shunt quantification and intracardiac flow assessment by fCMR.

## The safety of fetal MRI

The American College of Obstetrics and Gynecology (37, 38) and the American College of Radiology (39) support the use of MRI for fetal anomalies when ultrasound-based imaging is inadequate. Fetal MRI and fCMR do not generally use contrast-based agents, like gadolinium. There are theoretical risks of MRI during pregnancy, including fetal heating and energy deposition in fetal tissue, as well as acoustic noise. These risks are increased as magnet strength increases. Data has been limited to small populations, single institutions, and short-term follow-up. Nonetheless, there has been no reported adverse events, with more data on 1.5 T scanners compared to 3 T. Specifically, there is no difference in the incidence of congenital anomalies, neoplasm, fetal growth retardation, or vision or hearing loss in children exposed to MRI in-utero compared to controls (40, 41). Furthermore, compared to 1.5 T, fetal exposure to 3 T does not increase the risk of neurodevelopmental impairment (42). Recent data has shown promise using low-field MRI (0.55 T) for fetal imaging that may provide similar diagnostic quality images with lower specific absorption rate, low acoustic noise, and real-time imaging capabilities (43).

## Clinical utility of fetal MRI

### Extracardiac anomalies

Fetal MRI has been used over the last 30 years as an adjunct to ultrasound to define extra cardiac structures. This technology is useful in extracardiac pathology in the thorax including congenital lung malformations (44), congenital diaphragmatic hernia (45), and abdominal structures in heterotaxy (46), due to large field of view and excellent tissue contrast. In addition, fetal MRI has been shown to be useful to define extracardiac defects that impact the assessment of the heart such as identifying vascular tumors in fetuses with cardiomegaly or defining structural brain abnormalities in fetuses with concern for or confirmed genetic syndromes.

### Fetal MRI in CHD

The assessment of the fetal lungs by fetal MRI in CHD has been instrumental in understanding the presence of pulmonary lymphangiectasis in pulmonary venous obstruction from either primary pulmonary venous disease (total anomalous pulmonary venous return with obstruction) or restriction of the foramen ovale in hypoplastic left heart syndrome (HLHS) (47). Pulmonary lymphangiectasis, or the so-called “nutmeg lung,” has been associated with increased postnatal mortality and transplant (48). In tetralogy of Fallot with absent pulmonary valve, fetal MRI can be used to evaluate anatomic airway and lung findings that may be predictive of postnatal compromise from bronchial compression or congenital lobar emphysema (49). In fetuses with CHD, fetal MRI of the brain has been shown to be useful to define structural brain anomalies, brain volume, and brain injury that may impact neurodevelopmental outcomes (50, 51).

### Fetal CMR

Postnatal cardiac MRI is a common component of the pre-operative planning for many surgical procedures in the neonate. Similarly, subtleties in the cardiac anatomy can have major prognostic significance which may be useful in counseling prenatally. At present, the use of fCMR clinically is in its infancy. fCMR can clarify anatomy and physiology across a wide range of CHD. Prior studies have shown feasibility of performing fCMR in specific clinical scenarios including ectopia cordis (52), fetal arch anomalies (53), cardiac tumors (54), and other cardiac anomalies (55). In a retrospective study of 68 women carrying fetuses with CHD, 79% of the cases had agreement between fCMR and post-natal diagnosis (56). This is in comparison to 82% by fetal echocardiography. In fetuses with suspected coarctation by echocardiography compared to normals, fCMR revealed reduced ascending aorta flow and proximal displacement of the aortic isthmus in the third trimester to be highly predictive of postnatal neonatal critical coarctation. Thus, fCMR is a useful adjunct to help predict postnatal intervention (35).

In addition to anatomic clarification, the measurement of fetal cardiac axis, chamber size, ventricular function, and quantification of atrioventricular valve regurgitation is important for lesions with the potential for hydrops fetalis. These include extracardiac diagnoses like agenesis of the ductus venosus, twin to twin transfusion, cerebral arteriovenous malformations, pulmonary arteriovenous malformations, and sacrococcygeal teratoma. Primary cardiac abnormalities including fetal cardiomyopathy, fetal arrhythmias, cardiac tumors, Ebstein's anomaly and tetralogy of Fallot with absent pulmonary valve are at risk for worsening heart failure *in utero*. fCMR has been used successfully to quantitatively assess ventricular volumes, showing agreement with fetal echocardiography (57).

MRI can explain complex physiology using velocity-encoded phase contrast imaging. This conventional MRI approach can be used in fetal circulation to determine direction of blood flow through intracardiac shunts, cardiac output, and flow through the umbilical cord, ductus venosus, and cerebral artery (20). The impact of blood flow on the growth or lack of growth of cardiac structures may be better understood using 3D and 4D flow MRI. An extension of understanding flow distribution and velocity wave forms, is gaining insight into vascular resistance of various fetal vessels (11).

More granular details of fetal physiology involving oxygen transport and consumption have also been explored with MRI. Combining phase contrast imaging with T1 and T2 mapping to measure oxygen content, researchers can determine oxygen delivery and oxygen consumption (58). Cerebral oxygenation can be directly measured or reflected by the levels in the superior vena cava and aorta in CHD or cases of low cardiac output (59).

As fCMR becomes more available, expected appropriate referrals for fCMR in CHD will typically fall in three main categories. (1) extracardiac vascular structures, including aortic arch shape (60), (2) systemic and pulmonary venous anatomy (2), and (3) size of ventricles, either to monitor for ventriculomegaly or to gauge adequacy of biventricular physiology. Ventriculomegaly can be accurately assessed and serially monitored using CMR. Referrals for ventricular size assessment in prior studies have included cases of left sided obstruction, atrioventricular canal, and pulmonary atresia with intact ventricular septum (61). A list of potential use cases is presented in Table 1.

**Table 1:** Potential clinical use cases of fCMR.

• Coarctation (improving accuracy in diagnosis) • Vascular ring anatomy and effect on trachea/esophagus • Double outlet right ventricle (VSD classification and 3D anatomy of the outflow tracts) • Heterotaxy syndrome (venous anatomy, abdominal findings) • Detailed diagnosis of total or partial anomalous pulmonary venous drainage (including assessment of vertical vein) • Cardiomyopathy (assessment of myocardium and heart function) • Cardiovascular assessment of fetuses with hydrops fetalis • Cardiovascular assessment of fetuses with viral infections such as parvovirus or fetal HIV exposure • CHD with a borderline left heart to better determine left ventricular chamber size • Complex pulmonary artery blood supply including major aortopulmonary collaterals • Cardiac tumor characterization

## Conclusions

fCMR is a promising technology that can be used in conjunction with fetal echocardiography to improve accuracy of diagnosis, heighten sophistication of counseling and postnatal planning of CHD, and provide a comprehensive assessment of the cardiovascular system. Additionally, it may help cardiovascular surgeons and interventionalists plan for neonatal care in the fetal period. Data to support clinical insurance coverage for indications for fCMR are yet to be defined, though preliminary work suggests a role in both extracardiac vascular examination and intracardiac ventricular size and function. Research with phase contrast imaging, 4D flow, and T1 and T2 mapping may allow for a deeper understanding of fetal physiology including oxygen delivery and consumption in different forms of CHD and heart failure.

New technology, including DUS device enabling fetal cardiac gating, has allowed more centers to start performing fCMR. There are inherent technical challenges including achieving adequate spatiotemporal resolution and availability of trained staff and physicians to acquire, interpret, and communicate the imaging findings to the care team. Research in fetal electrocardiography may eventually allow for direct fetal cardiac gating, similar to postnatal CMR. With new technology in this rapidly advancing field, image, flow, and volume acquisition will be easier and more centers will obtain experience performing fCMR, allowing for it to be a more accessible and useful imaging tool in clinical care. Future research will help to delineate the clinical use of fCMR and effect on fCMR on long term outcomes of prenatally diagnosed CHD.
